# How accurately can we assess zoonotic risk?

**DOI:** 10.1371/journal.pbio.3001135

**Published:** 2021-04-20

**Authors:** Michelle Wille, Jemma L. Geoghegan, Edward C. Holmes

**Affiliations:** 1 Marie Bashir Institute for Infectious Diseases and Biosecurity, School of Life and Environmental Sciences and School of Medical Sciences, The University of Sydney, Sydney, Australia; 2 Department of Microbiology and Immunology, University of Otago, Dunedin, New Zealand; 3 Institute of Environmental Science and Research, Wellington, New Zealand; Princeton University, UNITED STATES

## Abstract

Identifying the animal reservoirs from which zoonotic viruses will likely emerge is central to understanding the determinants of disease emergence. Accordingly, there has been an increase in studies attempting zoonotic “risk assessment.” Herein, we demonstrate that the virological data on which these analyses are conducted are incomplete, biased, and rapidly changing with ongoing virus discovery. Together, these shortcomings suggest that attempts to assess zoonotic risk using available virological data are likely to be inaccurate and largely only identify those host taxa that have been studied most extensively. We suggest that virus surveillance at the human–animal interface may be more productive.

## Abstract 2

Determining which organisms harbour viruses that could potentially infect humans is of great topical interest. This Essay demonstrates that the data on which such zoonotic risk assessments are conducted are incomplete, biased, and rapidly changing with ongoing virus discovery.

## Introduction

Determining which animal species harbour viruses that could potentially infect humans is central to studies of disease emergence. It is therefore no surprise that studies performing “zoonotic risk assessment” have grown in popularity [1–5]. Although work of this type is often considered an integral part of pandemic preparedness, herein we argue that all such studies are severely impacted by the limited available data and hence may be of questionable value.

The vast majority of viruses remain to be described. Various estimates of the total number of viruses have been performed in the last decade [6,7]. For example, Anthony and colleagues surveyed the virome of the Indian flying fox (*Pteropus giganteus*) and from this extrapolated that the total number of mammalian viruses to be approximately 320,000 [8]. A more recent estimate suggests that mammals harbour 40,000 viruses, of which 10,000 may be zoonotic [7]. All these estimates are necessarily approximate, depend to some extent on how viruses are identified and defined, and do not account for the fact that viral lineages have rates of birth and death. Yet all agree that we have only sampled a tiny fraction of the virosphere.

Not only is our sampling of the virosphere extremely limited, but it is also strongly biased towards viruses of socioeconomic impact: those that impact human health, those in species we eat or keep as companions, and those that cause noticeable and major mortality events in domestic animals and wildlife. More recent large-scale virological sampling of wildlife using metagenomic next-generation sequencing has revealed an enormous number and diversity of novel viruses [9–12]. Total RNA sequencing, in particular, has massively increased the rate of virus discovery [13]. Even in host groups such as birds that have been surveyed for viruses for almost a century, new viruses continue to be described on a regular basis [14–16]. Consequently, our knowledge of viral diversity is rapidly expanding and far outstripping the rate at which the International Committee on Taxonomy of Viruses (ICTV) is able to classify virus species. Here, we argue that this change in sampling capability likely has major implications for assessing zoonotic risk, that viral discovery is still in its infancy, and that firm risk predictions based on current data remain premature.

### The extent and structure of virus data structure have changed markedly

Arguably the first publication that attempted to perform a risk assessment of aspects of disease emergence (rather than simply compiling lists of emerging viruses) was that of Jones and colleagues [1], which we use here as an arbitrary starting point. Additional studies have subsequently been published using a variety of techniques, yet all utilise clearly very limited data on the number and distribution of viruses in nature. These inherent data limitations are apparent when considering the dramatic change in the number of documented viruses that has occurred since the Jones and colleagues study. To illustrate this, we compared the change in (i) the number of viruses; (ii) the proportion of these viruses that are associated with disease; and (iii) the proportion that are known to be zoonotic (i.e., where either active infection or antibodies have been recorded in humans) between 2008 (the year of the Jones and colleagues study) and those available as of May 2020. We do so by examining 3 representative animal taxa: birds, fish, and shrews as an informative group of mammals. These taxa were chosen simply to illustrate the change in our sample of animal viruses, rather than being the species groups that are most likely to harbour zoonotic viruses. The methods used for data curation can be found in the Supporting information (S1 Methods), with the code and data available at https://github.com/jemmageoghegan/Assessing-zoonotic-risk.

Viruses in birds have been intensively studied for a century [17,18]. Despite this, our understanding of avian viruses is strongly biased towards those that cause disease in poultry and those with a known risk of zoonotic transmission, such as avian influenza A virus [19,20] and a number of vector-borne viruses [21,22]. Shrews are a mammalian group of increasing interest because they harbour a high number and diversity of viruses and often live close to human habitation. For example, shrews are important hosts for hantaviruses [23], although none directly associated with human disease, as well as a number of potential zoonotic viruses including coronaviruses [24] and Borna Disease Virus 1, a known zoonotic virus [25,26]. Despite this, shrews have generally been neglected in comparison to far more heavily studied taxa like rodents and bats. For example, 11 viral species have been identified in the most intensively studied shrew species—the common shrew (*Sorex araneus*). This is in comparison to 55 viral species described in a single study of Indian flying foxes [8] and 75 viral genera detected in Norway rats (*Rattus norvegicus*) [27]. Finally, although there are no examples of cross-species transmission from fish to humans, so they are not zoonotic hosts per se, our knowledge of fish viruses has accelerated with the significant economic impact of viral outbreaks in aquaculture [28,29], and hence they are useful indicators of our progress in virus discovery.

**Fig 1:**
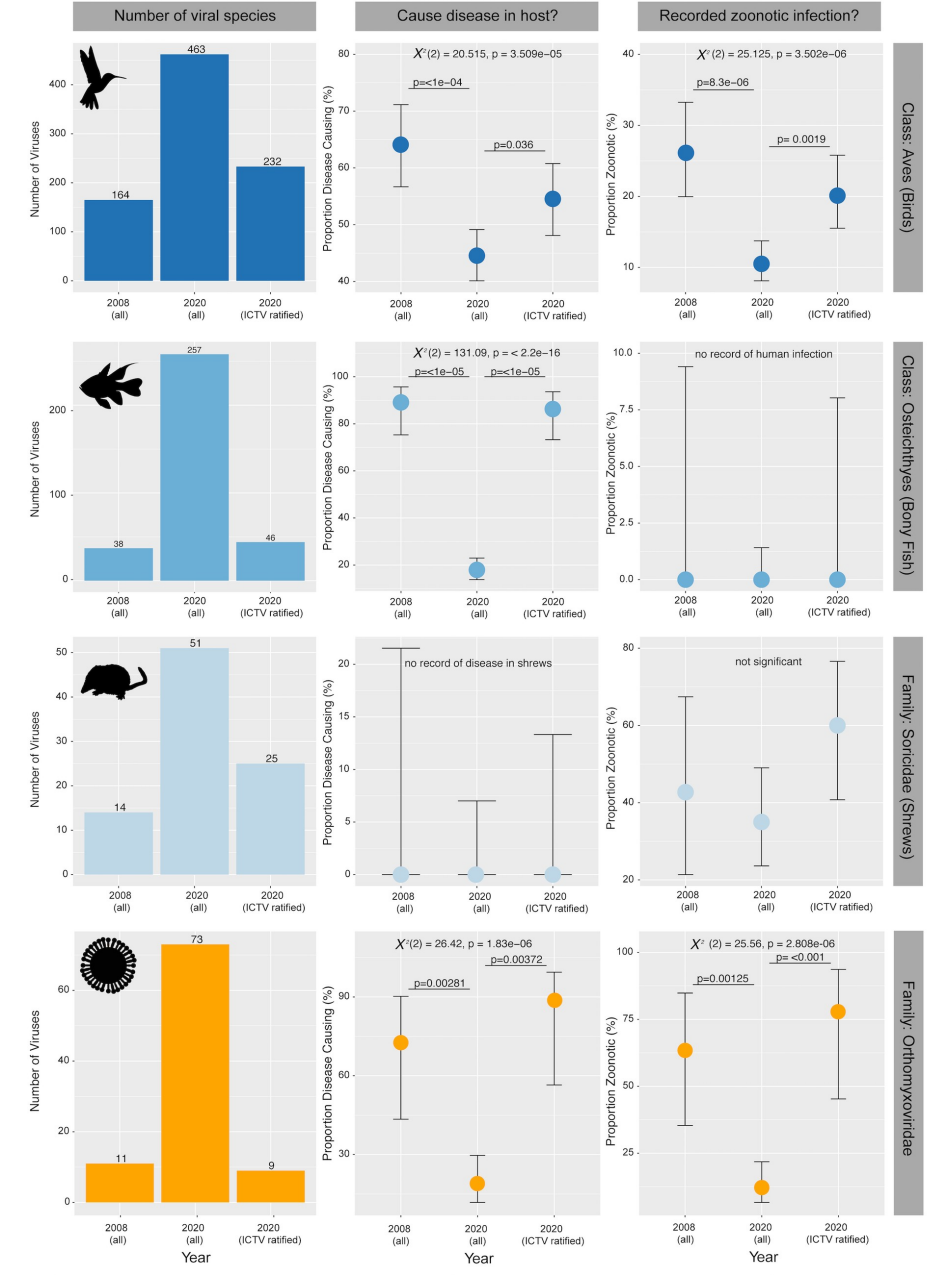
Change in the sampling of viral diversity in 3 host taxa and 1 virus family. Data are divided into 3 categories: (i) viruses described in 2008 regardless of ICTV status; (ii) viruses described in 2020 regardless of ICTV status; and (iii) viruses described in 2020 that have also been ratified as virus species by the ICTV. The first column shows raw counts of the number of viruses, with total virus counts shown above each bar. The second column shows the proportion of described viruses that are known to cause disease (from minor morbidity to mortality) in these hosts. The final column shows the proportion of viruses found in these taxa that have infected humans, identified through either virological or serological techniques (note that no fish viruses have been found to infect humans). Points are the proportional estimate (out of 100%), and bars are 95% confidence intervals. Statistics are shown in each plot, and *p-*values for post hoc tests are also shown. Animal silhouettes are from phylopic.org and distributed under a creative commons attribution. The virus silhouette was generated by M. Wille. The source code and underlying data for this figure can be found at https://github.com/jemmageoghegan/Assessing-zoonotic-risk.

Over the last decade, there has been a substantial increase in the number of viruses known to infect these hosts: a 10-fold increase in fish and almost a tripling in both birds and shrews (Fig 1). In addition, it is notable that the characteristics of these data have also changed. This is apparent as statistically significant differences in the proportion of viruses likely causing disease in their hosts and in the proportion that infect humans. In the case of birds, between 2008 and 2020, there has been a significant decrease in both the proportion of viruses that cause observable disease in these animals (*p* = 2.8 × 10^−5^) and the proportion of viruses for which human infection has been described (*p* = 8.3 × 10^−6^) (Fig 1). This shift is likely due to the discovery of more avian viruses in seemingly healthy wild birds [14,30,31]. We similarly see a significant difference in the proportion of viruses causing disease in fish, dropping from approximately 90% in 2008 to <20% in 2020 (*p* = 2 × 10^−16^) (Fig 1). Similar to birds, this is likely due to the large diversity of novel viruses described in fish markets and wild caught fish with no overt signs of illness [9,32,33]. This number is expected to decline further with continued sampling. For shrews, there was no statistically significant difference in the proportion of zoonotic viruses in this host between 2008 and 2020. This likely reflects continued and intensive research on hantaviruses [34–36] that are relatively commonplace in shrews.

Notably, these trends were also apparent in individual viral families. For example, the family *Orthomyxoviridae* (negative-sense RNA viruses) has been intensively studied as it contains both human influenza A and B viruses and undoubtedly represents a major zoonotic risk. Between 2008 and 2020, the number of orthomyxoviruses (and the related “orthomyxo-like” viruses) increased 7-fold (Fig 1). The composition of these data has also changed dramatically, with a huge decrease in the proportion of orthomyxoviruses that cause disease in their hosts (*p* = 0.00281) as well as the number of zoonotic viruses (*p* = 0.00125).

### Virus species ratified by the ICTV underestimate viral richness

A number of studies that assess zoonotic risk understandably rely only on those virus species that have been officially ratified by the ICTV or other databases that rely on ICTV data (e.g., RefSeq, https://www.ncbi.nlm.nih.gov/refseq/). Critically, for all 3 animal data sets, the number of virus species ratified by the ICTV in 2020 (i.e., the 2019 update) is substantially lower than the total number of viral species provisionally reported in these hosts from genomic studies. Indeed, the number of viral species ratified by the ICTV in 2020 is not significantly different from the total number of viruses described in 2008 for all host taxa. Hence, studies using only ICTV data are intrinsically out of date, in part because the sequences used to informally define new viruses and perform phylogenetic analysis in the literature are too short to be considered by the ICTV. As a result, large numbers of viruses described in the literature will likely never be ratified by the ICTV. While this is obviously a useful quality control, it is also limiting for studies attempting to extrapolate evolutionary characteristics from current virus diversity. As a case in point, one study [5] that relied on ICTV data analysed 66 avian viruses, only about 14% of the total number of viruses known to infect birds and hence is likely biased against those viruses recently sampled in apparently healthy animals and that are not associated with zoonotic infection. Strikingly, although they are unlikely to be zoonotic, most fish, and all invertebrate and amphibian viruses, including those described in some large-scale metagenomic studies [9,10], are not yet ratified. Also of importance was that we observed statistically significant differences in the proportion of disease-causing viruses (birds: *p* = 2.8e-05, fish: *p* < 2e-16) and those that are zoonotic (birds: *p* = 0.022) (Fig 1) in the 2020 and ICTV ratified data sets.

The disparity between the number of viruses described and those species ratified by the ICTV is also apparent in intensively studied viral families such as the *Orthomyxoviridae*. In this case, there is a 7-fold difference between the number of ICTV ratified orthomyxoviruses and those described in the literature. Since those viruses ratified by the ICTV are strongly focused on human and disease-causing animal viruses, this likely has major implications for our understanding of the proportion of disease-causing or zoonotic viruses. Using data from the most recent ICTV update is therefore a source of significant under-sampling and perhaps intrinsic bias.

### Study effort is inconsistent, but discovery rate is increasing

**Fig 2:**
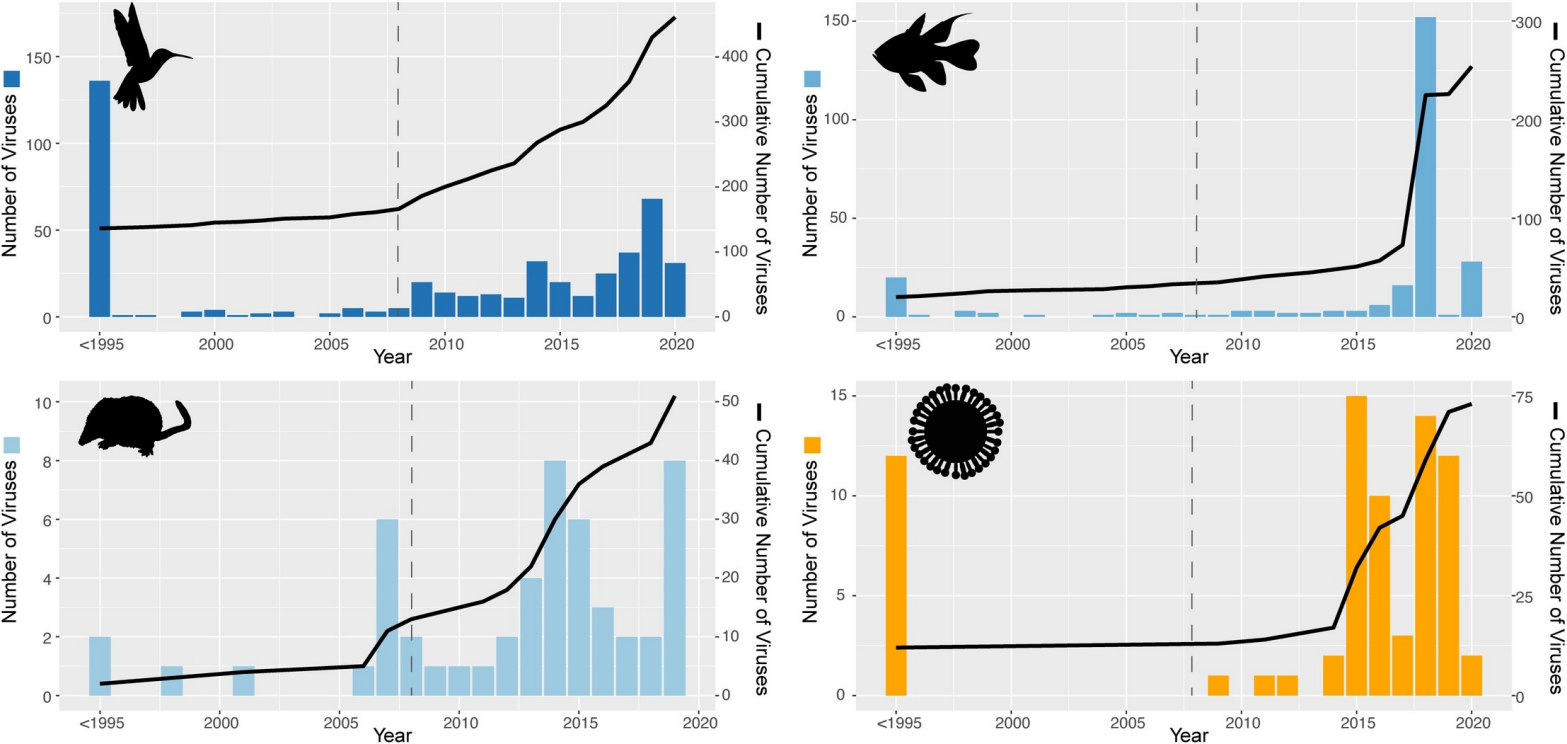
Recent increases in documented virus discovery. Bars indicate the number of new viruses described per year. Line represents the cumulative number of viruses described. All viruses described before 1995 have been presented as a single bar and thus the year is indicated as “<1995.” The dashed line indicates 2008, the year the Jones and colleagues paper [1] was published, and our cutoff for Fig 1. Animal silhouettes are from phylopic.org and distributed under a creative commons attribution. The virus silhouette was generated by M. Wille. The source code and underlying data for this figure can be found at https://github.com/jemmageoghegan/Assessing-zoonotic-risk.

The pace of virus discovery has greatly accelerated due to the rise of metagenomic, particularly total RNA sequencing. In birds, for example, 3 recent publications focusing on Australian waterbirds identified 42 new viral species, representing approximately 10% of all avian viruses described in the last century (*n* = 462) [15,16,30]. The average discovery rate in these publications was 80%, that is, 80% of viruses revealed were novel species. This is remarkable given that viruses have been described in poultry for 90 years: Indeed, by 1995, over 100 avian viruses had been described, all of which were poultry viruses, vector borne, or viruses causing overt disease in wild birds (Fig 2). Furthermore, these 3 studies only focused on the most highly sampled avian taxa (the orders Anseriformes and Charadriiformes) as they are important hosts for avian influenza A virus. Data on viruses that infect fish and shrews have similarly increased rapidly, with virus discovery rates of 86% and 75%, respectively, between 2008 and 2020 (Fig 2). Data from the *Orthomyxoviridae* paint a similar picture. Prior to 1995, most viruses described in this family were zoonotic or caused disease in birds, mammals, or fish. In the last 10 years, there has been a steady increase in the number of novel orthomyxoviruses described, particularly in invertebrates, amphibians, and fish. It is unlikely that this rate of discovery will decline in the near future.

**Fig 3:**
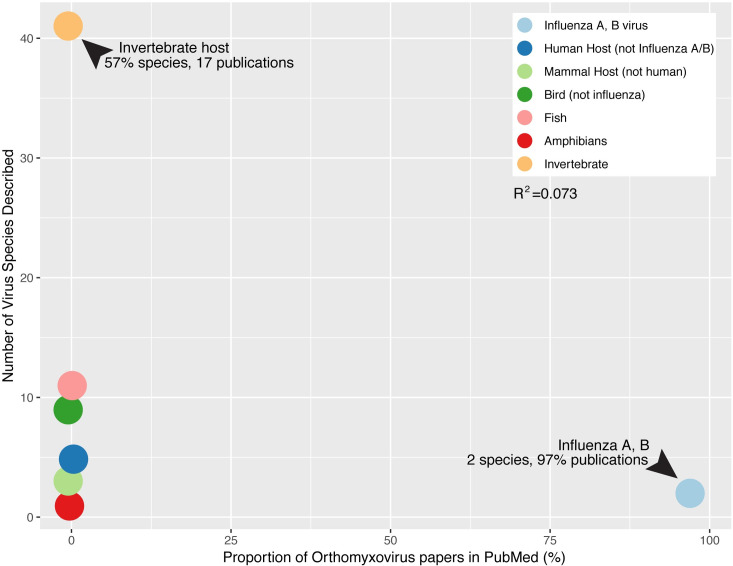
Disparate publication frequency between orthomyxoviruses that cause disease in humans or domestic animals (i.e., influenza A virus and influenza B virus) and those found in underappreciated vertebrate and invertebrate hosts. The source code and underlying data for this figure can be found at https://github.com/jemmageoghegan/Assessing-zoonotic-risk.

We also observed notable differences in virus discovery rates among host groups. As noted above, viruses that cause disease in humans necessarily garner more attention than other host species. For example, in the *Orthomyxoviridae*, some 97% of papers consider only 2 viral species: influenza A virus and influenza B virus (Fig 3). Viruses in other well-studied hosts within birds and mammals that are not zoonotic or do not cause morbidity or mortality are far less studied. For example, Johnston Atoll virus, a vector-borne virus of seabirds first described in 1963 [37,38], is only discussed in 7 publications indexed in PubMed. Despite strong evidence for large virus diversity in under-sampled hosts, more than 50% of the diversity of the orthomyxoviruses has been described in only 17 publications (approximately 0.03% of orthomyxovirus papers in PubMed) (Fig 3). A key issue is then whether this discrepancy in research effort creates biased data sets from which to estimate zoonotic risk. For example, there is a significant relationship between the number of viral species and the number of zoonotic viral species in a particular host [5], illustrating the intensive viral discovery efforts directed towards specific hosts and specific viral families. Although this particular sampling bias is often corrected for in zoonotic risk assessments, how well these corrections work is unclear. Hence, rather than predicting from which host taxa viruses are more likely to emerge, there is a concern that many studies may simply be demonstrating which host taxa have been studied most extensively.

### Limitations of metagenomics for assessing zoonotic risk

Metagenomic sequencing is now the main method used to identify novel viruses and as such plays a central role in studies aimed at assessing zoonotic risk. Despite its capacity for virus discovery, a major limitation of many metagenomic studies is that many lack information on the variables that are central to assessing zoonotic risk, including virus prevalence and host range. Indeed, a shortcoming of many metagenomic studies is that they identify sequences corresponding to a virus in 1 host species at 1 location, with very little downstream work undertaken done to screen large cohorts of animals for newly described viruses. This is particularly severe when the viruses identified do not cause observable disease or are found in animals of limited economic value. For example, because it secondarily reflects the ability of a virus to infect multiple species, host range is an important predictor of whether a virus may be zoonotic [2]. However, there is little information on the host range for the vast majority of viruses [2,8], unless the viruses in question are already known to be zoonotic. For example, Huaiyangshan banyangvirus (previously known as severe fever with thrombocytopenia syndrome virus and recently changed to Dabie bandavirus) was first described in 2011, and due to the severity of human infections, there has been intensive screening of animals to understand its reservoir hosts [39,40], with the virus being identified in both shrews and birds [40]. This is in marked contrast to other viruses, such as numerous hantaviruses described in shrews, most of which have only been described on a single occasion. For example, Kilimanjaro Virus [41], Qian Hu Shan Virus [42], Azagny Virus [43], Lena River Virus [44], and Sarufutsu Virus [45] have been described once, and there is no evidence in the literature of follow-up studies to determine the host range, ecology, or epidemiology of these viruses.

Metagenomic studies may also lead to uncertain or even incorrect host associations. This is particularly apparent in studies using faecal, cloacal, or gut samples in which the viruses detected may actually be present in the host diet and microbiome, rather than viruses that actively replicate in the animal of interest. For example, members of the Picobirnaviridae recovered from avian cloacal samples have been referred to as vertebrate viruses [30,44,45], although more recent analyses suggests that they may in fact be associated with bacteria [46]. Fortunately, tools are being developed to help assign viruses to hosts, some of which extend phylogeny-based studies [47], although are still obviously constrained by the very limited number of viruses available for analysis.

### Methods for zoonotic risk assessment

There is understandably a desire to develop more robust and meaningful ways to assess the zoonotic risk posed by different viruses. Our aim here is not to critically evaluate the different models being used to evaluate zoonotic risk assessment, but rather to reveal the significant biases and shortcomings in the data upon which these models rely. An array of statistical models has been developed to assess zoonotic risk, each attempting to deal with inherit biases in the available data, although few consider that these data likely represent less than 1% of possible vertebrate viruses. As outlined by Becker and colleagues [48], approaches employed in zoonotic risk assessment fall into 2 categories. First, trait-based approaches predict zoonotic reservoirs or vectors based on building profiles utilising specific characteristics (such as morphology, ecology, and phylogeny) and rank potential zoonotic reservoirs that fit the same profile [49]. These models can achieve “out of sample” prediction, that is, they are able to predict host species for which there are currently no known viral associations provided they have a profile that is similar to species for which vial associations are known. However, because they rely on extrapolating profiles, they are likely prone to output existing patterns of observed host–pathogen data. Second, network-based approaches attempt to estimate unobserved host–virus interactions based on an observed network of associations, comprising pairs of hosts and associated viruses. These methods only allow for the prediction of host species for which viral data already exist and favour species for which a large diversity has already been described [48]. As a consequence, these methods may tend to favourably predict livestock (and potentially bats and rodents) as likely hosts [3,4].

Beyond the current literature, the recently developed “Spillover” tool (https://spillover.global/) estimates zoonotic risk of viruses from a variety of host, environmental, and viral factors [50]. “Spillover” integrates features of the host (epidemiology, ecology, and genetics), the environment, and the virus (genetics, epidemiology, virology, and ecology), with risk scores calculated using both data analysis and expert opinion. Although the insights generated may sometimes be informative, the utility of all such approaches is likely to be impacted by major data limitations unless the major sampling biases described here are accounted for.

For many viruses, only “patchy” data are currently available, which may be so limited as to include only a single description: In these circumstances, it is challenging to accurately integrate any features of the host, environment, or virus on which to make a prediction, again limiting the applicability of tools like “Spillover.” Consider, for example, the ranking of members of the Coronaviridae. There are a number of coronaviruses ranked for which there are only limited sequence data, such as “BtVs-BetaCoV/SC2013” (rank 41) or “coronavirus PREDICT CoV-35” (rank 21). To date, there is a single full genome and a single partial genome for BtVs-BetaCoV/SC2013 [51,52], making it unclear how features like host range or virus epidemiology are inferred. In some cases, such as coronavirus PREDICT CoV-35, a number of sequences are available but all are partial (200 to 400 bp) and derived from a single study [51]. It is therefore likely that this virus was ranked without available empirical data for many of the relevant variables.

In sum, despite ongoing work in developing statistical models used for zoonotic risk assessments, we contend that the underlying data are often incomplete and will likely result in biased and inaccurate predictions.

### Sampling the animal–human interface

We have argued that assessments of zoonotic risk are often based on background data that are themselves hugely limited in quantity, intrinsically biased, out of date, and hence are likely inaccurate. For example, it is only recently that shrews were recognised as important host taxa, and beyond rodents, shrews, and voles, we know little about the viruses of small mammals or marsupials. Similarly, pangolins were systematically ignored as virus reservoirs, although it is now clear that these animals are infected by a number of viruses [53], including those closely related to Severe Acute Respiratory Syndrome Coronavirus 2 (SARS-CoV-2) [54].

Given the enormity of the virosphere, that RNA viruses evolve so quickly that repeat sampling will be regularly required to accurately track natural diversity, and that virome composition will likely vary across the geographic range occupied by an individual host species, a more targeted, and arguably more productive, approach will be to focus sampling directly at the animal–human interface [55]. For example, immunological studies at the bat–human interface have already identified bat SARS-related coronaviruses with the potential to infect humans [56] that should be prioritised for surveillance. Beyond bats and the people living around bat roosts, humans working in poultry production, piggeries, abattoirs, and live animal markets, those participating in animal hunting and slaughter for bushmeat, as well as the animals they interact with, should be targeted for both immunological and metagenomic surveillance. This will provide a baseline understanding of the viral diversity in these potential hosts and a meaningful real-time and empirical estimate of the frequency of virus spillover between animals and humans, rather than an estimate based on biased and incomplete data. Currently, such surveillance efforts are limited to particular viral species, such as influenza A virus, for which cross-species transmission between animal and humans is well established [57,58]. There is undoubtedly scope for interrogating the animal–human interface more broadly, by targeting both human communities exposed to wild animals based on where they live or work [59,60] and the animals they interact with.

It might also be profitable to focus on the frequency which viruses are found in multiple hosts, indicative of spillover and hence potential emergence, within a specific ecosystem. In such cases, sampling intensity could be structured according to the population density of the vertebrate species present, as more dense populations are also likely to harbour the highest diversity of microbial taxa [61]. We believe that examining the frequency at which viruses jump between host species on such epidemiological (i.e., short term) timescales will offer more actionable insights than the predominantly phylogeny-based studies performed so far that can only infer rates of cross-species transmission on long-term evolutionary timescales [2] and have little relation to tempo of disease emergence. Given hypothesised differences in rates of virus spillover in pristine compared to highly disturbed environments [62,63], this type of study could be performed at forest fringes, enabling us to better understand the contribution of environmental factors, including changing land, use to disease emergence.

Although the risk assessment of viruses or hosts with zoonotic potential in theory enables enhanced surveillance strategies and will aid pandemic planning, we have clearly sampled a both a minute and highly biased fraction of the virosphere. In the same way as identifying the exact animal origin of SARS-CoV-2 is a needle-in-the-haystack task, so predicting which of the myriad of animal viruses might emerge in humans is like finding a particular grain of sand on a beach. Herein, we have demonstrated both the ever-changing nature of the underlying data and the significant data limitations and biases that are currently incorporated into zoonotic risk assessments. Hence, despite the sophisticated analytical methods employed, risk assessments may be less accurate than currently appreciated. We argue that the time has come to accept the major data limitations that underpin those zoonotic risk assessments performed to date and to turn to more detailed and targeted real-time virome sampling at the animal–human interface.

## Supporting information

S1 Methods Materials and methods. (DOCX)
