# Supplementary material for: How accurately can we assess zoonotic risk?
Source: PLoS Biol. 2021 Apr 20;19(4):e3001135. doi: 10.1371/journal.pbio.3001135 (PMC8057571; doi:10.1371/journal.pbio.3001135)
Supplement: S1 Methods — (DOCX) [file pbio.3001135.s001.docx]

**How accurately can we assess zoonotic risk?**

Short title: Limitations of zoonotic risk assessment

Michelle Wille^1^*, Jemma L. Geoghegan^2,3^, Edward C. Holmes^1^

Methods supplement

Materials and Methods

We collated (May 2020) all viral species (both DNA and RNA) from birds (Class Aves; n = 462), fish (Class Osteichtyes and Cyclostomata; n = 258) shrews (Family Scorcidae; n = 51). Viruses were identified through publication searches using both keyword searching and interrogating reference lists and citation lists of relevant articles, using the NCBI taxonomy browser for descriptions of viral species in families known to infect the host taxa in question (<https://www.ncbi.nlm.nih.gov/Taxonomy/Browser/wwwtax.cgi>), and similarly analysing ICTV (<https://talk.ictvonline.org/taxonomy>) and associated ICTV reports (2018/2019 updates).

For those virus species ratified by the ICTV, we used their species descriptions. Accordingly, if multiple viruses were classified as a single species, we reported the virus as a single species. For viruses that are not ratified ICTV species, we scanned the articles describing them and used the reported phylogenies and pairwise comparisons with consideration of the cut-off thresholds used previously (1) to distinguish between viral species and inter-species variants. Accordingly, viruses were considered as different species if they exhibited <90% sequence similarity in the RdRp protein or <80% across the whole virus genome.

We included retroviruses that were obviously exogenous, but excluded endogenous retroviruses with no evidence of an exogenous phase. We also included arthropod-borne viruses, or viruses transmitted by tick, flea and mosquito vectors as long as there was evidence that they infected vertebrate hosts. In the case of metagenomic studies, we excluded viruses that were unlikely to cause infection in their hosts (such as those part of the microbiome or diet) by removing viral families or genera that have only been recorded to infect plants, fungi, invertebrates and bacteria.

To better understand the composition of these data sets, we scored each virus species across four categories: (i) whether the virus had been described in 2008 (i.e. the year of the Jones et al. paper (2)). Viruses for which no genetic sequence was generated prior to 2008, but had been described phenotypically, were included in this category; (ii) whether these virus species were ratified by the ICTV (as of May 2020); (iii) whether there were reports of viruses causing disease in their hosts (i.e. morbidity – in birds this includes subtle morbidity such as egg drop in layers – or mortality) through scanning the available literature; and (iv) whether there has been any recorded infection in humans, either though active infection or from serology studies. In cases where a single viral species contained many well-described viruses, we score this species as causing disease even if disease was only observed in a single virus.

We used a similar approach to collate all members of the *Orthomyxoviridae* (n = 73), including all documented “orthomyxo-like” viruses (1, 3). Due to the large discrepancy between the number of ICTV ratified orthomyxoviruses and those described in publications, we relied heavily on data provided in the latter (e.g. refs(1, 4, 5) for taxonomic assignment). For viruses that were described in 2008, but classified in a different viral family, we scored this category as a “N”. For example, Upolu virus and Aransas Bay virus were previously included in the *Bunyavirales* but reclassified as *Orthomyxoviridae* in 2014 (6). Publication counts for well-described orthomyxoviruses were mined from PubMed using generic search terms such as “influenza A virus”. For orthomyxoviruses from invertebrates and lower vertebrates we carefully searched for publications on PubMed, particularly those from Shi et al. (1, 5) , and through GenBank sequence entries to ensure we captured all publications.

For each host taxonomic group, we aimed to reveal any difference in the proportion of disease-causing and zoonotic viruses in 2008 (regardless of ICTV status), 2020 (regardless of ICTV status) and only those virus species ratified by the ICTV in 2020 (2019 update). To this end, we used generalized linear models (glm, family = binomial) followed by Tukey posthoc testing (glht) using the *multcomp* package. Statistics were performed using R 3.5.3 (7) and figures were generated using the *ggplot2()* package.

Curated data and Rmarkdown file are available at: <https://github.com/jemmageoghegan/Assessing-zoonotic-risk.git>.

References

1. Shi M, Lin XD, Tian JH, Chen LJ, Chen X, Li CX, Qin XC, Li J, Cao JP, Eden JS, Buchmann J, Wang W, Xu JG, Holmes EC, Zhang YZ. 2016. Redefining the invertebrate RNA virosphere. Nature 540:539-543.

2. Jones KE, Patel NG, Levy MA, Storeygard A, Balk D, Gittleman JL, Daszak P. 2008. Global trends in emerging infectious diseases. Nature 451:990-994.

3. Wille M, Holmes EC. 2020. The ecology and evolution of influenza viruses. Cold Spring Harb Perspect Med 10.

4. Li CX, Shi M, Tian JH, Lin XD, Kang YJ, Chen LJ, Qin XC, Xu J, Holmes EC, Zhang YZ. 2015. Unprecedented genomic diversity of RNA viruses in arthropods reveals the ancestry of negative-sense RNA viruses. Elife 4:e05378. doi: 10.7554/eLife.05378.

5. Shi M, Lin XD, Chen X, Tian JH, Chen LJ, Li K, Wang W, Eden JS, Shen JJ, Liu L, Holmes EC, Zhang YZ. 2018. The evolutionary history of vertebrate RNA viruses. Nature 556:197-202.

6. Briese T, Chowdhary R, Travassos da Rosa A, Hutchison SK, Popov V, Street C, Tesh RB, Lipkin WI. 2014. Upolu virus and Aransas Bay virus, two presumptive bunyaviruses, are novel members of the family Orthomyxoviridae. J Virol 88:5298-309.

7. R Development Core Team. 2008. R: a language and environment for statistical computing, R Foundtation for Statistical Computing, Vienna, Austria. <http://www.R-project.org>.
